# The Role of Immune-Related miRNAs in the Pathology of Kidney Transplantation

**DOI:** 10.3390/biom11081198

**Published:** 2021-08-12

**Authors:** Emanuela Boštjančič, Željka Večerić-Haler, Nika Kojc

**Affiliations:** 1 Institute of Pathology, Faculty of Medicine, University of Ljubljana, 1000 Ljubljana, Slovenia; emanuela.bostjancic@mf.uni-lj.si; 2 Department of Nephrology, University Medical Centre, 1000 Ljubljana, Slovenia; zeljka.vecerichaler@kclj.si; 3 Faculty of Medicine, University of Ljubljana, 1000 Ljubljana, Slovenia

**Keywords:** microRNA (miRNA), kidney transplantation, immune response

## Abstract

MicroRNAs (miRNAs) are members of the non-coding regulatory RNA family that play pivotal roles in physiological and pathological conditions, including immune response. They are particularly interesting as promising therapeutic targets, prognostic and diagnostic markers due to their easy detection in body fluids and stability. There is accumulating evidence that different miRNAs provide disease-specific signatures in liquid samples of distinct kidney injuries. Using experimental models and human samples, there have been numerous suggestions that immune-related miRNAs are also important contributors to the development of different kidney diseases as well as important markers for monitoring response after kidney transplantation. However, there are limited data for understanding their function in the molecular pathways of allograft pathologies. In our review, we focused on microRNAs that are related to different aspects of immune response after kidney transplantation.

## 1. Introduction

### 1.1. MicroRNAs

MicroRNAs (miRNAs) are short regulatory RNAs that act as posttranscriptional repressors of gene expression regulating physiological and pathological conditions, including immune response [1]. Detailed description on their biogenesis is beyond the scope of current review and can be found elsewhere [2].

Briefly, the importance of miRNAs could be observed through ablation of the components of their biogenesis or silencing co-factors that results in severe embryogenesis defects in every animal studied [2]. Perhaps the best evidence that miRNAs are important for normal physiological functions comes from experiments in which components of the miRNA biogenesis pathway are depleted or overexpressed, e.g., DGCR8 as an essential co-factor of the RNAse II enzyme Drosha, the reduced enzymes Dicer and Drosha, and the overexpression of Dicer, Ago 2 and Exportin-5 [3].

Different mechanisms of regulation of miRNA biogenesis are used, not only to generate the spatiotemporal specificity of miRNA, but also to achieve the necessary levels and dynamics of expression. There is a variety of silencing complexes, functional complex of miRNAs and proteins, which repress mRNA translation, that produce different molecular effects on their targets. Multicellular organisms have taken advantage of this variability in the outcome of miRNA-mediated repression, using them differently in specific cell types or even in different subcellular compartments [2]. Many miRNAs are expressed in a tissue-specific manner, e.g., *miR-208* is cardiac specific [4], *miR-122* is liver specific [5] and/or cell type specific, e.g., *miR-223* is primarily expressed in granulocytes [2]. In repressing translation of mRNAs, miRNAs act in different ways under normal cell conditions. For mRNAs that should not be expressed in a particular cell type, miRNAs reduce protein production, resulting in silencing of the targets. In another way, miRNAs can adjust protein production, leading to tailored expression in certain cell types but more uniform levels in other cell types, serving as a fine-tuning of target expression. Another mechanism of miRNAs is that they can act as a bystander, where downregulation by miRNAs is tolerated or negated by feedback processes, resulting in neutral target expression [6].

Canonical and non-canonical mechanisms in miRNA biology, their specific expression and variations influence different developmental stages and physiology of multicellular organisms. Disruption of these mechanisms can lead to undesirable pathogenesis of various diseases [2]. miRNA functions are mainly postulated by in vivo experiments, through the phenotypic consequences of a mutated miRNA or an altered mRNA complementarity site, both of which can disrupt miRNA regulation. In some cases, function has been inferred from the effects of transgenic constructs leading to ectopic expression of miRNA [6]. miRNAs are important in the regulation of morphogenesis and maintenance of undifferentiated or incompletely differentiated cell types, such as in stem cell differentiation, cardiac and skeletal muscle development, neurogenesis, haematopoiesis, control of cell fate decision, cell proliferation, cell death, neuronal patterning, modulation of hematopoietic lineage differentiation, and control of the timing of developmental transitions [2,3,6]. Under physiological conditions, miRNAs are involved in metabolism, the regulation of insulin secretion, cholesterol metabolism, resistance to viral infections and oxidative stress, immune response, etc. [7]. Therefore, each cell type at each developmental or physiological stage might have a different miRNA expression profile. It is believed that miRNA biogenesis and function is one of the most important regulatory mechanisms in maintaining tissue identity during embryogenesis and adult life [2].

### 1.2. miRNAs’ Contribution to Disease Pathogenesis—Causes of Aberrant Expression of miRNAs

miRNAs are associated with various pathological conditions due to the disruption of miRNA biogenesis or its complex that results in dysregulation of miRNAs, leading to inappropriate expression and consequently function [8]. An abnormal miRNA expression profile is usually the consequence of their regulation by aberrantly expressed transcription factors, genomic rearrangements, alterations in genes encoding miRNAs, or epigenetic mechanisms. All of this could contribute to inappropriate regulation of protein-coding genes and ultimately to the development of disease, e.g., genetic disorders, cancer, autoimmune and inflammatory diseases and neurodegenerative and cardiovascular diseases [2,8].

Disruption of miRNA-target interaction in the form of single nucleotide polymorphisms (SNP), both in the miRNA gene or its target site in the mRNA, can lead to a complete gain or loss of miRNA function, thereby establishing a disease state [9]. In contrast to miRNA target sites in mRNA transcripts, where the potential for variation is very large (e.g., *AT1R* and *miR-155)* [9], the variants identified in miRNA precursor sequences are extremely rare (e.g., *miR-196* [10]). The presence of SNP in pri-miRNA or pre-miRNA can also affect the processing of miRNAs and their expression, which can also lead to different disease outcomes [11].

Aberrant methylation or de-methylation of any gene (protein-coding or protein-non-coding) can lead to repression and silencing of a physiologically activated gene or de-repression of a physiologically silenced gene, respectively. In the case of miRNAs, this mechanism leads to the inappropriate expression of miRNAs, either de-methylation and expression of miRNAs that should be silenced or methylation and silencing of miRNAs that should be expressed. All of these aberrant expressions can be the cause of disease development [12].

## 2. miRNAs in Kidney Diseases and in Immune Response Including Kidney Transplantation

### 2.1. miRNAs in Kidney Diseases

Based on experimental models and human samples, there is ample evidence that miRNAs also play important roles in the development of various kidney diseases, suggesting that aberrant miRNA regulation might be a cause of disease. There is an increasing evidence that different miRNAs represent disease-specific signatures not only in tissue samples but also in liquid samples from different kidney injuries. Moreover, since these molecules are stable and can be easily detected in body fluids, such as blood and urine, they are even more interesting and promising as potential prognostic and diagnostic markers for non-invasive monitoring of various diseases [1].

miRNAs have been shown to play a role in a broad spectrum of urinary tract diseases, ranging from various tumors, infectious diseases, systemic (auto)immune diseases with renal involvement to isolated immune-related renal diseases [13], which not only cause native renal damage, but can sometimes recur after kidney transplantation [14]. There are numerous suggestions that miRNAs are important regulators of all these outcomes, becoming new class of promising prognostic biomarkers [1].

### 2.2. miRNAs in Immune Response Including Kidney Transplantation

Solid organ transplantation is a highly immunologically complex process associated with the need to optimize donor-recipient MHC (major histocompatibility complex) antigen matches to achieve graft acceptance. Many different components of the immune system are involved in graft tolerance or rejection, including antibodies, antigen-presenting cells, subsets of helper and cytotoxic T-cells, immune cell surface molecules, signaling mechanisms, and cytokines [15]. The development of pharmacological agents that interfere with the alloimmune response has been instrumental in the success of organ transplantation, leading to improved graft survival, lower doses of necessary, albeit toxic, immunosuppressive drugs, and better long-term outcomes for patients. Immune response plays a crucial role in the most common complications that may occur after allograft transplantation, i.e., rejection and non-rejection complications (e.g., antibody-mediated rejection, T-cell-mediated rejection, delayed graft function, viral infections, drug tox-icity, systemic diseases, recurrent or de novo glomerular diseases, etc.). miRNAs are involved in all aspect of immunity, from inflammation and innate to adaptive [16].

## 3. Signature of Immune-Related miRNAs Dysfunction and/or Expression after Kidney Transplantation

Kidney biopsy remains the gold standard for diagnosing renal transplant complications, i.e., rejection and non-rejection complications. Evidence that miRNA expression within the graft and liquid samples (serum, plasma, urine) predicts renal graft status, suggests that noninvasively determined miRNA profiles may in the future minimize the need for invasive biopsy procedures and predict the development of acute rejection and chronic allograft nephropathy [17]. Although there is ample evidence that miRNAs are important markers for monitoring response after kidney transplantation, there are limited data for understanding their function in the molecular pathways of allograft pathologies [1].

In our review, we focused on miRNAs associated with various aspects of the immune response after kidney transplantation that were recognized as promising diagnostic or prognostic marker. The specific signature might not be the consequence of the induction of transcription factors only but also be due to changes in methylation status and the presence of single nucleotide polymorphisms (SNPs). The majority of publications have analyzed changes in the expression pattern, but only a few have analyzed the methylation status and the presence of SNPs among the genes for miRNAs.

### 3.1. Kidney Transplant Rejection

Allograft rejection remains a major problem in solid organ transplantation, as it can lead to either acute or chronic loss of graft function. Acute rejection comprising T-cell mediated rejection and antibody-mediated rejection is an immune process that begins with the recognition of the allograft by the immune system as a non-self, and untreated progresses to chronic rejection with the destruction of the graft [17]. The rejection process may be clinically silent or associated with varying degrees of worsening of allograft function and urinary abnormalities such as proteinuria. The gold standard for the diagnosis of graft rejection remains renal biopsy, where histological lesions are thoroughly evaluated according to the Banff criteria, which are mainly used for research purposes to assess various aspects of T-cell-mediated and antibody-mediated rejection, as well as acute versus chronic active rejection [18]. Recently, molecular approaches, including the molecular microscope diagnostic system (MMDx) [19], have been developed to improve the diagnosis of rejection. Since renal biopsy is an invasive procedure, noninvasive biomarkers such as miRNA detected in serum/urine would be of great importance in the diagnosis, particularly early subclinical rejection.

### 3.2. Aberrations on DNA Level

#### 3.2.1. Single Nucleotide Polymorphism (SNP)

One of the most well-known miRNA involved in innate immune response is *miR-146a* [20]. In contrast, *miR-196a* was mainly associated to cancerogenesis and tumor progression [21], but few studies also described its involvement in different aspects of immune response [22]. Both miRNAs were investigated for the presence of SNPs in the genes for miRNAs, *miR-146a* G > C (rs2910164) and *miR-196a-2* C > T (rs11614913), and for an association with kidney transplant rejection. In samples from 100 kidney transplant recipients of Iranian ethnicity, it was found that the CC genotype, C and G alleles of *miR-146a* G > C were associated with an increased risk of rejection when comparing patients with rejected to non-rejected kidney transplants. The CC genotype, T and C alleles of *miR-196a-2* C > T were also significantly more common in transplanted patients compared with healthy controls, but there were no significant associations to kidney transplant rejection. The SNPs of *miR-146a* G > C and *miR-196a-2* C > T may be genetically susceptible factors for graft rejection and the development of renal disease, respectively [23]. Further studies are needed to validate the involvement of these two SNPs in patients of other ethnicities as predictive and prognostic markers.

#### 3.2.2. Methylation

One of the main causes of chronic kidney disease of the native and transplanted kidney is the progressive fibrosis of the interstitium and the accompanying tubular atrophy that lead to renal failure. Some authors have suggested that one of the causes may be also in the DNA methylation patterns. In a study on kidney biopsy samples from kidney transplant recipients 24 months after transplantation, the role of DNA methylation and miRNAs on graft gene expression was investigated. The study showed that methylated sites were strongly enriched in regions of high tissue specificity, while the methylation pattern corresponded with gene expression data and both were related to immune response (activated state) and nephrogenesis (inhibited state). Epigenetic modifications may thus influence the expression, not only protein-coding genes, but also of immune-related miRNAs that contribute to development of interstitial fibrosis, graft function and inter-individual variation in long-term outcomes [24].

### 3.3. Dysregulation of Human miRNAs Expression and Allograft Dysfunction after Kidney Transplantation

Alterations in miRNA expression can influence or contribute to the final outcome after renal transplantation. The majority of analyses addressing this issue have been performed with blood samples (blood cells and/or plasma/serum), and only a limited number have examined the expression of miRNAs within the graft and/or in correlation with mRNAs. Table 1 summarizes the current knowledge about the expression of immune-related miRNAs in different complications after kidney transplantation.

#### 3.3.1. Stable Graft Function without Immunosuppression

In a study of Danger et al., the expression profiles of miRNA in peripheral blood mononuclear cells (PBMCs) was analyzed. PBMCs were obtained from operationally tolerant patients (patients with withdrawal of the immunosuppression) and from patients with stable graft function and treated with conventional immunosuppression. Overexpression of *miR-142-3p* was showed in B cells in the operationally tolerant patients; retrospective analysis showed that the expression of *miR-142-3p* was stable over time and was not modulated by immunosuppression. Overexpression of *miR-142-3p* modulated hundreds of genes associated with the B-cell immune response, including potential *miR-142-3p* targets and molecules previously identified in the blood of immunotolerant patients. *miR-142-3p* expression in B cells may thus contribute to the maintenance of tolerance [29] and could potentially serve as a prognostic marker for immunosuppression withdrawal. This study further confirmed the regulation of immune-response related processes by *miR-142-3p* [36] also in immune-tolerance to received graft.

#### 3.3.2. Acute Rejection

Changes in miRNA expression in allografts regulate the expression of genes involved in adaptive immunity and thus acute rejection. Understanding the regulatory interplay of relevant miRNAs and mRNAs in the rejecting allograft will lead to a better understanding of the molecular pathophysiology of alloimmune injury [20]. Furthermore, novel, accessible biomarkers of acute rejection can more easily allow us to detect rejection earlier and enable finer tailoring of immunosuppressive or new target treatment [23]. miRNAs and mRNA profiles in urine and peripheral blood cells have already been associated with acute kidney allograft rejection [17].

One of the first studies analyzed miRNAs in kidney allograft biopsies with acute rejection and without complications and used them as a training and validation set. A strong association was found between intra-graft expression of miRNAs and mRNAs. Acute rejection and renal allograft function can be predicted with a high degree of precision using intra-graft levels of miRNAs. Expression analysis of miRNAs showed that *miR-142-5p*, *miR-155* and *miR-223*, which are well-known regulators of the immune cells, are overexpressed not only in biopsies from patients with acute rejection but are also highly expressed in PBMCs. There is likely a cellular basis for the altered intra-graft expression of miRNAs, suggesting the utility of miRNA expression patterns as biomarkers of human kidney allograft status [30]. In another study, mRNA and miRNA expression was profiled in allograft biopsies with and without acute rejection. For acute rejection, specific dysregulations of both miRNAs and mRNAs were identified: 1035 mRNAs that correlated with nine relevant miRNAs and were regulated by p53 and FoxP3. There is a highly regulated interplay between specific mRNA/miRNAs in allograft rejection, which influences immune-mediated injury during acute rejection. Infiltrating lymphocytes contributed to tissue dysregulation of miRNA in case of rejection. The expression of many of these miRNAs was significantly associated with the intensity of interstitial inflammation and tubulitis [37], further supporting the role of identified miRNAs in innate immunity after kidney transplantation.

#### 3.3.3. Antibody Mediated Rejection

Antibody-mediated rejection (AMR) is an important cause of allograft dysfunction and loss after organ transplantation. It is associated with the generation of donor-specific antibodies, which bind to human leukocyte antigen (HLA), or non-HLA molecules expressed on endothelial cells within the renal allograft [38]. AMR can be both acute and chronic, and studies suggest that miRNAs may contribute to this type of rejection. Expression profiling of miRNAs in PBMC of kidney transplant recipients with chronic AMR or stable graft function identified 257 expressed miRNAs. Among 10 selected miRNAs, immune-related *miR-142-5p* was, again, increased not only in PBMC but also in biopsies from patients with chronic AMR as well as in the experimental model of chronic AMR. It was further observed that *miR-142-5p* is not modulated in PBMCs of patients with renal insufficiency; these results suggested that its overexpression in chronic AMR is the consequence of the immunological disorders rather than of the renal dysfunction. Moreover, its expression was also not modulated in PBMC of patients with acute rejection and was not affected by treatment, confirming previous results. *miR-142-5p* was thus identified as a potential biomarker that allows very good discrimination of patients with chronic AMR from others. Finally, analysis of predicted *miR-142-5p* targets showed enrichment of immune-related genes [31]. These results may improve the understanding of the mechanisms of chronic rejection.

Damage of glomerular capillaries during AMR may lead to deleterious inflammation and fibrosis and this damage may be also due to changes in expression of miRNA in glomerular capillaries that is poorly understood. A set of 16 promising candidate miRNAs were identified in a glomerulo-endothelial in vitro model of AMR. The selected miRNAs were validated in micro-dissected glomeruli from human transplant biopsies compared with matched controls without evidence of AMR. Twelve of these 16 glomerulo-capillary miRNAs were either upregulated (*let-7c-5p*, *miR-28-3p*, *miR-30d-5p*, *miR-99b-5p*, *miR-125a-5p*, *miR-195-5p*, *miR-374b-3p*, *miR-484*, *miR-501-3p*, *miR-520e*) or downregulated (*miR-29b-3p*, *miR-885-5p*). This identified miRNA signature could improve our understanding of AMR and be useful for diagnostic or therapeutic purposes [34].

#### 3.3.4. T-Cell Mediated Rejection

In kidney allografts, T-cell-mediated rejection is characterized by infiltration of the interstitium and tubules by T-cells and macrophages. The potential of using miRNAs as biomarkers of severe T-cell-mediated vascular rejection in kidney transplant patients was revealed by analyzing their expression in PBMC of kidney transplant recipients. Initial analysis revealed 23 differentially expressed miRNAs that distinguished patients with T-cell-mediated rejection from patients with stable grafts. Of these, further validation of expression was performed for six differentially expressed miRNAs in patients with T-cell-mediated rejection, AMR, interstitial fibrosis/tubular atrophy (IF/TA), stable graft function, and urinary tract infection. The expression levels of all six candidate miRNAs were significantly downregulated in the PBMC of patients with T-cell-mediated rejection compared with the other groups and showed high sensitivities and specificities for the diagnosis of T-cell-mediated rejection, and the combination of five miRNAs even increased this sensitivity and specificity. The combined measurement of *miR-15a*, *miR-16*, *miR-103a*, *miR-106a*, and *miR-107* may thus help to identify T-cell-mediated rejection after kidney transplantation more accurately and be clinically applicable [25]. There are limited data on the expression of miRNA in chronic active T-cell-mediated rejection; however, all five potentially diagnostic miRNAs were already shown to be involved in immune response [39].

#### 3.3.5. Interstitial Fibrosis/Tubular Atrophy (IF/TA)

Progressive IF/TA of various etiologies is a major cause of chronic allograft dysfunction and loss after transplantation [13]. A noninvasive diagnosis of IF/TA would be useful [26]; therefore, miRNAs were investigated as promising biomarkers. Biopsy samples from patients with IF/TA and from patients with normal allografts showed significant upregulation of *miR-142-5p* and *miR-142-3p* and significant downregulation of *miR-211* in kidney allograft tissues from patients with IF/TA compared with those with normal allografts. In addition, the same trend of the expression of *miR-142-3p* and *miR-211* in the PBMC samples was confirmed. Simultaneous differential expression of miRNAs in renal allograft biopsy and in PBMC in patients with IF/TA suggest that expression of miRNAs in PBMC could be used as potential non-invasive biomarker of renal graft function. *miR-142-5p* in the biopsy samples and *miR-142-3p* and *miR-211* in both the biopsy and PBMC samples could be used as diagnostic biomarkers for IF/TA and as predictive factors for allograft function [16]. In addition to *miR-142, miR-211* is also well accepted immune-modulatory miRNA [40]. Several candidate miRNAs in plasma exosome or whole plasma in comparison to kidney biopsy have been also investigated as biomarkers of IF/TA in patients after kidney transplantation. The expression of these miRNAs from plasma exosomes or total plasma with different severities of IF/TA did not differ from stable graft function. In kidney biopsy samples, *miR-21*, *miR-142-3p*, and *miR-221* were higher in biopsies with a high fibrosis score than in samples with a lower score. Moreover, high expression of *miR-21* in the exosome but not in total plasma could differentiate the degree of fibrosis, although there was no correlation between *miR-21* level in exosome and current standard biomarkers of renal damage. High *miR-21* content in plasma exosome but not in total plasma thus indicate high-grade IF/TA in patients after kidney transplantation [26]. *miR-21* is the type of multi-functional miRNA that has a wide spectrum of functions, including the modulation of the immune system [41]. Another study examined the expression levels of four immune-related miRNAs (*miR-21*, *miR-31*, *miR-142-3p*, and *miR-155*) in plasma samples from kidney recipients with either long-term stable allograft function or biopsy-proven IF/TA, or healthy controls. The expression of *miR-21*, *miR-142-3p* and *miR-155* was significantly upregulated in plasma samples from patients with IF/TA compared to the groups with stable allograft function and healthy controls without kidney disease. Plasma *miR-21* concentration was negatively correlated with creatinine and positively correlated with the estimated glomerular filtration rate. It was found that *miR-21*, *miR-142-3p* and *miR-155* in plasma samples were able to discriminate most patients with IF/TA from the other two groups, suggesting their diagnostic potential for renal dysfunction and graft monitoring [27]. As well as *miR-21*, *miR-155* is multi-functional miRNA; however, its major role is in regulating immune response of antibody producing cells and fibrosis [42].

#### 3.3.6. Nephrotoxicity

Calcineurin is involved in many aspects of renal development and function. Potent immunosuppressants include calcineurin inhibitors such as cyclosporine A and tacrolimus, which may also have nonimmune effects. They can cause nephrotoxicity associated with tubulo-interstitial fibrosis and inflammation, which affects up to half of all transplant patients [35]. Calcineurin inhibitors induce nephrotoxicity by poorly understood mechanisms, limiting their use in transplantation and other diseases [43]. Regarding the treatment of kidney transplant recipients, the majority of research analyzes miRNAs in patients treated with cyclosporine A, but there are limited data about the involvement of immune-related miRNAs in patients treated with tacrolimus.

In animal studies of cyclosporine-induced nephrotoxicity, an early increase in *miR-494* expression was observed. In vitro studies confirmed an upregulation of *miR-494* and a decrease in the level of its target protein PTEN, leading to the activation of cyclosporine-induced epithelial-to-mesenchymal transition (EMT). Knockdown of *miR-494* prevented the downregulation of *PTEN* in tubular epithelial cells after cyclosporine treatment and prevented cyclosporine-induced EMT. Manipulation of *miR-494* expression might have therapeutic potential as a novel approach to prevent EMT that is associated with cyclosporine-induced nephrotoxicity [32]. Besides the regulation of EMT, *miR-494* is also involved in the regulation of macrophage polarization, thus influencing immune system activation [44]. Further studies identified a miRNA–mRNA interaction map, allowing exploration of the role of miRNAs in cyclosporine-induced nephrotoxicity and the gene pathways they regulate. Cyclosporine causes specific changes in miRNAs and mRNAs associated with the silencing complex, the complex that is crucial for miRNA function, suggesting that they alter post-transcriptional regulation of gene expression. Next-generation sequencing of total RNA revealed that only a fraction of all miRNAs and mRNAs are actively inserted into the silencing complex. A role for miRNAs in calcineurin-independent regulation of *JNK* and *p38* through targeting of *MAP3K1* was revealed. Ingenuity pathway analysis of canonical pathway targeted by miRNAs in cyclosporine-treated cells suggest final influence on inflammatory and anti-inflammatory genes [43]. Furthermore, cyclosporine was shown to induce significant changes in renal miRNA expression profiles when experimental animals receiving cyclosporine were compared to those not receiving it. Seventy-six differentially expressed miRNAs were identified, including miRNAs associated with renal fibrosis, e.g., *let-7d*, *miR-21*, *miR-29*, *miR-30*, *miR-130*, *miR-192*, and *miR-200*, as well as miRNAs not reported to be associated with nephrotoxicity or immunosuppression in vivo. Pathway analysis of miRNA–mRNA alterations highlighted *Wnt*, *TGF-β*, *mTOR*, and *VEGF* signaling pathways and among biological pathways there were endocytosis and T-cell receptor signaling pathway. The in vivo and in vitro results correlated with each other, and the miRNAs and mRNAs tested as directly related to cyclosporine treatment were *miR-21*, *miR-186* and *miR-709*, as well as *BMPR1a*, *SMURF1* and *SMAD7*. These data provide a basis for investigating the role of known and novel candidate miRNAs in the mechanism of nephrotoxicity and their further therapeutic potential [35]. Most of these identified miRNAs were already shown to be involved in certain aspect of immune system modulation and/or activation [45].

#### 3.3.7. Ischemia-Reperfusion (IR) and Acute Tubular Injury

Ischemia-reperfusion (IR) injury is also a major challenge in organ transplantation. An imbalance of metabolic supply and demand within the ischemic organ results in profound tissue hypoxia and microvascular dysfunction. Subsequent reperfusion enhances the activation of innate and adaptive immune responses and cell death programs. Recent advances in understanding the molecular and immunological consequences of IR may lead to innovative therapeutic strategies for the treatment of patients with IR-associated tissue inflammation and organ dysfunction [46]. IR frequently occurs in renal transplantation and is associated with acute kidney graft injury. *miR-30* has been found to stimulate the immune response and reduce inflammation in renal IR. Animal experiments and in vitro experiments showed that *miR-30c-5p* (among other targets) inhibited the expression of CD86 (M1 macrophage marker) and promoted the expression of CD206 (M2 macrophage marker). *miR-30c-5p* may reduce renal IR by converting M1 macrophages into M2 macrophages and lead to changes in inflammatory cytokines [28].

Delayed graft function can be one of the complications after renal transplantation, which can be caused by numerous conditions, including acute tubular injury. Acute tubular injury develops in several phases, all accompanied by different immune mechanisms, from necroinflammation and tissue damage in the early phase to functional recovery of renal function in the recovery phase, with a profound contribution of immune cells [47]. Our unpublished data on the expression of immune-related miRNAs in acute tubular injury further supports the involvement of miRNAs regulation of immune mechanisms in the transplanted kidney.

#### 3.3.8. Bacterial Infections

One of the most common complications in renal transplant recipients are urinary tract infections, mainly caused by uropathogenic Escherichia coli. Immunosuppressant cyclosporine activates the cells that are a preferential site of adhesion and translocation for Escherichia coli. Cyclosporine induces inhibition of lipopolysaccharide-induced activation of cells by downregulating the expression of TLR4 via *let-7i*. Using an experimental model, it was shown that pretreatment with cyclosporine before infection induced a marked decrease in cytokine production, neutrophil recruitment, and a dramatic increase in bacterial load, but not in infected TLR4-defective kidneys of the experimental models. In vitro and in vivo models thus confirmed that the use of an anti-let-7i during kidney transplantation can protect cyclosporine-treated patients from bacterial infection [48]. This miRNA may represent a potential therapeutic target in immune response to bacterial infection in kidney transplant recipient.

### 3.4. Expression of Viral miRNAs after Kidney Transplantation

In addition to human-encoded miRNAs, viral-encoded miRNAs can also influence ghost immune response and changes in host miRNA expression after renal transplantation. While it is difficult to distinguish between the viral and human origins of certain miRNAs, some of them are either human- or viral-specific [1].

#### 3.4.1. Epstein–Barr Virus

The Epstein-Barr virus was the first human virus found to express miRNAs. Circulating Epstein-Barr viral miRNA profiles were generated in pediatric kidney transplant patients who had overcome Epstein-Barr viral infection or had chronically high viral loads in comparison to non-transplant patients with acute infectious mononucleosis. Plasma *ebv-miR-BART2-5p* was present in a higher number of patients with infectious mononucleosis and with chronic high viral loads than in those who had resolved Epstein-Barr viral infection. The same trend was observed between the number of miRNAs of Epstein-Barr virus expressed in plasma and viral load. Several ebv-miRs were detected, including *ebv-miR-BART7-3p*, *ebv-miR-15*, *ebv-miR-9-3p*, *ebv-miR-11-3p*, *ebv-miR-1-3p*, and *ebv-miR-3-3p* only in patients with infectious mononucleosis and with chronically high viral loads. Lytic *ebv-miRs-BHRF1-2-3p* and *ebv-miR-1-1*, indicating active viral replication, were detected only in patients with infectious mononucleosis. These results suggest that *ebv-miR-BART2-5p*, which targets the stress-induced immune ligand MICB to evade recognition and elimination by natural killer cells, may play a role in maintaining high Epstein-Barr viral loads in pediatric kidney transplant recipients with chronic high viral loads [49]. Analysis of expression of viral-specific miRNA might thus represent a marker for monitoring of disease stage of EBV-infected kidney transplant recipients.

#### 3.4.2. BK Polyomavirus

BK polyomavirus infection is a common asymptomatic viral infection in the general population. However, in immunocompromised individuals, such as polyomavirus-associated nephropathy in kidney transplant recipients, severe complications have been observed [50]. Polyomavirus BK encodes two mature miRNAs—*bkv-miR-B1-3p* and *bkv-miR-B1-5p*—that regulate the viral life cycle [51]. Information on miRNA expressions of BK polyomavirus is limited, although they control viral replication and contribute to immune evasion. Expression of these two viral miRNAs was quantified and detected in the vast majority of plasma samples from nine patients with polyomavirus-associated nephropathy and two controls. On average, a ninefold higher expression level was detected in patients with polyomavirus-associated nephropathy compared to controls. Further results suggest that viral miRNA expression is increased in polyomavirus-associated nephropathy patients also in the presence of rearranged viral strains The frequent detection and increased expression of miRNAs suggest involvement in the pathogenesis of polyomavirus-associated nephropathy [50]. In contrast, *bkv-miR-B1-5p* and *bkv-miR-BJ1-3p* were measured in paired preserved plasma and urine samples from kidney transplant recipients with either early- or late-stage infections. All patients had abundant *bkv-miR-B1-5p* and *bkv-miR-BJ1-3p* in urine, but not in plasma. Their higher urinary level was associated with higher urinary viral loads, but with no significant difference between early- and late-stage infection [51]. Another study in culture systems investigated the autoregulatory and immunomodulatory effects of these two miRNAs; however, there are limited data from clinical samples [52].

#### 3.4.3. Human Cytomegalovirus

Human cytomegalovirus infections are common after renal transplantation and can be detected directly by viral DNA or indirectly by the host immune response. Human cytomegalovirus also encodes miRNA, which can alter the pathobiology of cytomegalovirus infections and contribute to disease progression. Viral miRNAs can be detected in blood, but it was shown in a recent study that they can also be detected in saliva samples. Namely, the miRNAs *miR-UL112-5p*, *miR-US5-2-3p*, *miR-UL36*, *miR-US25-2-3p* and *miR-UL22a* encoded by human cytomegalovirus were detected in the saliva of fifteen kidney transplant recipients and three healthy controls, with *miR-US5-2-3p* being the most frequently detected. The presence of human cytomegalovirus miRNAs is associated with an increased T-cell response to cytomegalovirus infection in kidney transplantation, suggesting an association with frequent reactivations of human cytomegalovirus [53]. The detection of cytomegalovirus in saliva would represent a quick and the non-invasive method of detecting CMV infection.

## 4. Therapeutic Opportunities of Immune-Related miRNAs

### 4.1. miRNAs as Therapeutic Targets

miRNAs as small molecules make their delivery in vivo feasible. The use of chemically modified oligonucleotides either to target a specific miRNA or disrupt miRNA-mRNA binding may lead to the inactivation of pathological miRNA [54,55]. For under-expressed miRNA, the re-introduction of the mature miRNA into the affected tissue would restore regulation of the target gene. For this purpose, artificial miRNAs have been developed (miRNA mimic) to enhance the expression of beneficial miRNAs or the introduction of short hairpin duplex, similar to pre-miRNA, into the cell. This suggests that individual miRNAs, which are a potential therapeutic marker, can be targeted to appropriate tissue. Most of the developed protocols have used local administration in easily accessible tissue; systemic delivery also has some promising results; tissue and cell-type-specific targeting remains the major challenge [3,54,55].

Anti-sense oligonucleotides (ASOs) are short and single-stranded antisense oligonucleotides and, in the context of miRNA inhibition, are called anti-miRNA oligonucleotides (AMOs). Over-expressed miRNA can be down-regulated by reducing the mature miRNA level through direct targeting (mature miRNA, pri-miRNA or pre-miRNA) or by reducing the components of miRNA biogenesis. Chemically engineered oligonucleotides, termed “antagomirs”, have been developed and proven to be efficient and specific silencers of endogenous miRNAs in mice. Chemical modifications and cholesterol conjugations stabilize and facilitate intravenous delivery of antagomirs. They interact with miRNAs in the cytoplasm and lead to specific miRNA down-regulation when injected systemically or locally [3,54,55]. In another approach, miRNA sponges have been developed to inhibit several miRNAs; miRNA sponges possess multiple binding sites and could be useful for sequestering a miRNA family. Furthermore, miR-masks and miR-erasers have also been developed; an miR-mask has been designed for masking the miRNA binding site on target mRNA, whereas an miR-eraser is similar to miR-sponges, except that the miR-eraser uses only two copies of the antisense sequence. Gene-specific miRNA mimic and miRNA-masking antisense approaches have been used to test the possibility of using miRNAs and their corresponding targets as therapeutic targets [56,57].

We need more knowledge concerning which miRNAs to target, how to produce and stabilize them, and how to direct them to the organ. The specificity of drug-like oligonucleotides is important because of the off-target effect. The off-target effect is also a significant challenge, especially considering that miRNA-mediated repression often requires a homology of only six to seven nucleotides in the seed region of the miRNA and mRNA target site. Toxicity due to chemical modifications, which is used to facilitate cellular uptake and prevent degradation, should be taken into account [3,54,55]. Many studies have investigated the delivery of miRNAs or their inhibitors to the target site using extracellular vesicles [58].

### 4.2. Potential Use of the Extracellular Vesicles for miRNA Mimic and/or Inhibitors Delivery

Extracellular vesicles are also promising as biomarkers, but even more interesting as therapeutic tools in renal transplantation [59]. The detailed structure, function and potential use of extracellular vesicles is beyond the scope of this review and is summarized in detail elsewhere [60]. In summary, extracellular vesicles are among other functions responsible for crosstalk between graft tissues and the innate/adaptive immune system, and play an important role in allorecognition, ischemia-reperfusion injury, autoimmunity and alloimmunity. Extracellular vesicles are known to have an immunomodulatory role in renal transplantation. They transmit graft antigens, costimulatory/inhibitory molecules, cytokines, growth factors and functional miRNAs that can modulate the expression of recipient cell genes. Extracellular vesicles from neutrophils and macrophages act as immunosuppressive and immune-stimulatory factors on dendritic cells throughout the body. In contrast, extracellular vesicles from dendritic cells mediate alloantigen spread and modulate antigen presentation to T-lymphocytes. Based on the biogenesis of extracellular vesicles, they can enhance complement activation or secrete complement inhibitors and prevent cell lysis. Platelet-derived extracellular vesicles affect pro-coagulation and pro-thrombosis, while endothelial cell-derived extracellular vesicles promote endothelial survival and angiogenesis after ischemic injury and, together with tubular-derived extracellular vesicles, contribute to ischemia-reperfusion injury and healing. They may also induce rejection by inducing both alloimmune and autoimmune responses. Endothelial extracellular vesicles also have procoagulant/proinflammatory effects and may release self-antigens leading to tissue-specific autoimmunity. Extracellular vesicles from the renal tubule transport pro-fibrotic mediators (TGF-β and *miR-21*) to interstitial fibroblasts, leading to peritubular capillary rarefaction and interstitial fibrosis/tubular atrophy. Extracellular vesicles derived from mesenchymal stromal cells are considered to be a promising therapeutic agent in experimental models of rejection and ischemia-reperfusion injury. They are able to inhibit apoptosis and inflammatory fibrogenesis, induce autophagy to protect tubular and endothelial cells, and stimulate tissue regeneration by triggering angiogenesis, cell proliferation, and migration [59]. As such, extracellular vesicles represent an excellent delivery method for potential therapeutic miRNAs.

The therapeutic use of extracellular vesicles would be a real advantage compared to mesenchymal stem/stromal cells MSCs that have been extensively studied because of their potential therapeutic role. The use of extracellular vesicles would avoid potential immune rejection and improve safety. Moreover, mesenchymal stem cells release extracellular vesicles containing relevant biomolecules such as miRNAs, but translation of their use into the clinic is still in the early stages. A number of concerns remain to be addressed but most preclinical studies have been successful, e.g., a protective role in acute kidney injury after ischemia reperfusion, modulation of both innate and adaptive immune responses in graft-versus-host disease [58]. Furthermore, Massa et al. (2020) investigated the mechanism of extracellular vesicles derived from immature dendritic cells in regulating T-cell differentiation and immune tolerance, including plasma cytokine levels associated with rejection. They found that these dendritic cells significantly improved the percentage survival, decreased the inflammatory response, reduced CD4+ T-cell infiltration, and reduced cytokines associated with rejection. It was found that *miR-682* was highly expressed in immature dendritic cells and modulate the sub-population of CD4+ T cells. ROCK2 was identified as a target of *miR-682* [33].

## 5. Conclusions

miRNAs as other non-coding regulatory RNAs play pivotal roles in physiological and pathological conditions, including the immune response. Using experimental models and human samples, there have been numerous suggestions that immune-related miRNAs are not only important contributors to the development of different kidney diseases but are also important markers for monitoring response after kidney transplantation.

Kidney biopsy still represents the gold standard for diagnosing renal transplant complications. However, there are still numerous unresolved issues including what is the trigger for the rejection, giving importance to finding unexplored mechanisms in terms of non-coding RNAs including miRNAs. There is accumulating evidence that different miRNAs provide disease-specific signatures in tissue and liquid samples of distinct kidney injuries after kidney transplantation, representing promising therapeutic targets, as well as prognostic and diagnostic markers, especially due to their easy detection in bodily fluids and stability.

## Figures and Tables

**Table 1:** Overview of human immune-related and differentially expressed miRNAs in complications after kidney transplantation ^1^.

miRNA	Complication	Sample Type	Level of Change	Marker Type	Reference
*miR-15a*, *miR-16*, *miR-103a*, *miR-107*	T-cell mediated rejection	PBMC	Down-regulation	Diagnostic	[25]
*miR-21*	IF/TA	PBMC, tissue	Up-regulation	Diagnostic	[26,27]
*miR-30c-5p*	Ischemia-reperfusion	In vitro, in vivo	Non applicable	Therapeutic	[28]
*miR-142-3p*	Acute rejection, IF/TA, immunosuppressive drugs withdrawal	PBMC	Up-regulation	Diagnostic, prognostic, predictive	[16,26,27,29]
*miR-142-5p*	Acute rejection, IF/TA	PBMC, tissue	Up-regulation	Diagnostic, prognostic	[16,30,31]
*miR-155*	Acute rejection, IF/TA	PBMC, tissue	Up-regulation	Diagnostic, prognostic	[27,30]
*miR-211*	IF/TA	PBMC, tissue	Down-regulation	Diagnostic, predictive	[16]
*miR-221*	IF/TA	PBMC, tissue	Up-regulation	Diagnostic, predictive	[26]
*miR-223*	Acute rejection	PBMC, tissue	Up-regulation	Diagnostic, prognostic	[30]
*miR-494*	Nephrotoxicity	In vitro, in vivo	Non applicable	Therapeutic	[32]
*miR-682*	Extracellular vesicles from dendritic cells	In vitro	Non applicable	Therapeutic	[33]
*let-7c*, *miR-28*, *miR-29b*, *miR-30d*, *miR-99b*, *miR-125a*, *miR-195*, *miR-374b*, *miR-484*, *miR-501*, *miR-520e*, *miR-885*	Antibody-mediated rejection	Tissue	Up-regulation, except *miR-29b* and *miR-885* that were down-regulated	Diagnostic, therapeutic	[34]
*Let-7d*, *miR-29*, *miR-30*, *miR-130*, *miR-186*, *miR-192*, *miR-200*, *miR-709*	Nephrotoxicity, Immune suppression	In vitro, in vivo	Non applicable	Mechanism of neurotoxicity, therapeutic	[35]
*let-7i*	Bacterial infection in cyclosporine treated patients	In vitro, in vivo	Non applicable	Therapeutic	[34]

^1^ Legend: IF/TA, interstitial fibrosis tubular atrophy; PBMC, peripheral blood mononuclear cells.

## Data Availability

Not applicable.
